# Esophageal remodeling in eosinophilic esophagitis

**DOI:** 10.1097/MOG.0000000000001031

**Published:** 2024-04-12

**Authors:** Anisa Shaker

**Affiliations:** University of Southern California, Keck School of Medicine of USC, Department of Medicine, Division of Gastrointestinal and Liver Diseases, Swallowing and Esophageal Disorders Center, Los Angeles, California, USA

**Keywords:** endothelial cells, Eosinophilic esophagitis, fibroblasts, fibrosis, Th2 cells

## Abstract

**Purpose of review:**

Eosinophilic esophagitis (EoE) is a Th2 immune/antigen-mediated disorder characterized by esophageal dysfunction and eosinophilic inflammation. Worsening dysphagia and food impactions are significant complications associated with esophageal remodeling and fibrostenotic disease. This review highlights the most recent research findings pertaining to mechanisms of sub-epithelial fibrosis in EoE, current diagnostic tools, and therapeutic approaches.

**Recent findings:**

Recent studies leveraging publicly available single cell sequencing databases and comparative proteomics have furthered our understanding of the mechanisms mediating fibrosis. Fibroblast crosstalk with the extracellular matrix and with epithelial, endothelial, and T cells have been implicated, with the likely existence of multiple fibroblast sub-types. Accurate diagnosis of remodeling with biopsies remains a challenge due to inadequate depth of sampling. Web-based tools incorporating epithelial findings show promise in predicting subepithelial fibrosis. Impedance planimetry with esophageal distensibility measurements are increasingly utilized tools to assess fibrostenotic severity. Immunostaining and luminal captured proteins associated with remodeling show promise as potential molecular markers of fibrosis. Anti-inflammatory therapy may improve esophageal fibrosis and distensibility, although specific fibrosis-targeted therapy is lacking.

**Summary:**

Recent studies highlight novel mechanisms of fibrosis in EoE. Improved understanding of these mechanisms may lead to novel diagnostic strategies and therapies, and thereby inform treatment decisions.

## INTRODUCTION

Eosinophilic esophagitis (EoE) is a Th2 immune/antigen-mediated clinicopathologic disorder characterized by symptoms of esophageal dysfunction (usually dysphagia) and an eosinophil predominant inflammation of ≥15 eosinophils per high power field in esophageal mucosal biopsies. Several studies support the prevailing theory that untreated, persistent inflammation (e.g., due to delays in diagnosis or therapy) can initiate tissue remodeling processes that involve epithelial hyperplasia, subepithelial fibrosis, and hypertrophy of the esophageal smooth muscle, progressing in many, though not all adults to a fibrostenotic phenotype with strictures and luminal narrowing [1–5]. Worsening dysphagia and esophageal food impactions requiring endoscopic interventions and need for esophageal dilations are the most significant complications and are associated with endoscopic features [6] and, to a certain extent, histopathologic features of fibrostenotic disease [7].

EoE is a patchy and transmural disease, and peak epithelial eosinophil counts may not be the best or only parameter of disease activity, and they may not reflect the degree of subepithelial activity [8^▪^]. Esophagectomy specimens from EoE patients show that deeper layers including the submucosa, muscularis propria, and adventitia are also affected [9]. A muscular variant of EoE has also been described in patients with hypercontractile esophageal motility disorders undergoing per oral esophageal myotomy with elevated subepithelial eosinophilia despite normal epithelial eosinophils [6]. This review highlights the most recent research findings pertaining to current diagnostic tools to assess remodeling, mechanisms of sub-epithelial fibrosis in EoE, and therapeutic approaches targeted to fibrosis. 

**Box 1:**
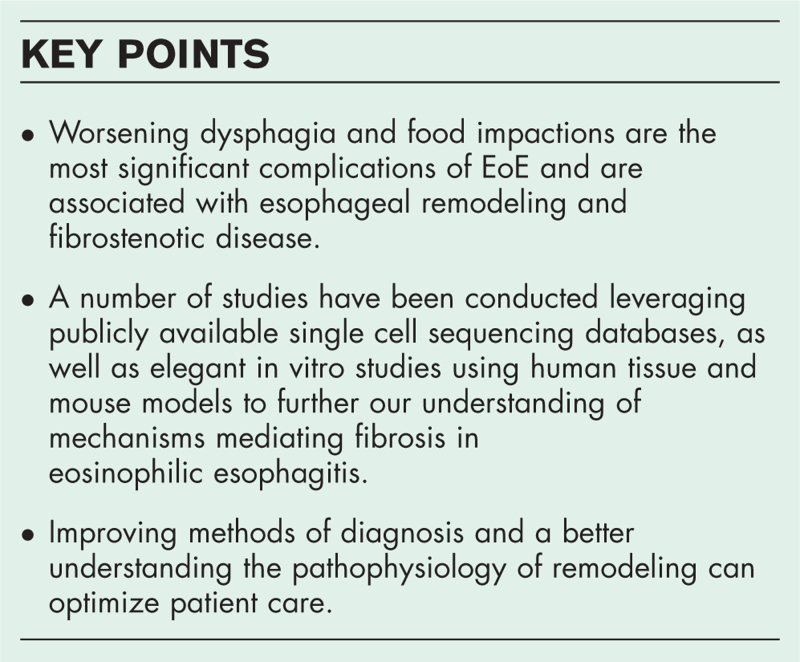
no caption available

## DIAGNOSIS OF REMODELING

Diagnosis of histopathologic fibrosis has proved challenging with most studies using standard forceps, whose biopsies consists mostly of epithelium that contains adequate lamina propria in only ∼50% of samples [10]. This observation has raised concern that esophageal biopsies using standard forceps assess sub-epithelial disease severity incompletely and inadequately in EoE [6]. Lamina propria sampling can be optimized with larger, specialized forceps that allow sampling of subepithelial tissue in >90% of biopsies [11]. Using this approach, one study of 200 EoE patients showed that correlations between subepithelial and epithelial peak eosinophil counts as well as endoscopic and symptom severity, while significant, were modest. There was also some discordance between epithelial and sub-epithelial findings, with biopsies of 40% of patients with < 15 intraepithelial eosinophils/hpf showing subepithelial peak counts of ≥15/hpf [7]. These findings suggest that while the clinical utility of histologic assessment of sub-epithelial alterations needs further investigation, such assessment may be useful in those patients who have ongoing symptoms but who do not fulfill current diagnostic cut-offs for EoE based on epithelial eosinophil counts alone. In addition, it is helpful to consider that lamina propria fibrosis can be an early feature of EoE, even in the absence of overt endoscopic findings such as esophageal stricture [12].

Improvement in lamina propria sampling, even with larger forceps may not be feasible/practical in clinical practice, however. Lamina propria fibrosis assessment across labs and studies can also be inconsistent. Therefore, a variety of approaches are being used to predict the presence of fibrosis in the lamina propria or deeper esophageal layers in EoE patients. Hiremath *et al.* have used web-based tools to develop and validate prediction models for lamina propria fibrosis in esophageal biopsies that have inadequate lamina propria, using the grade scores for surface epithelial alterations and dyskeratotic epithelial cells [13^▪^], which they had previously shown to be concordant with the presence of lamina propria fibrosis in children [14]. Surface epithelial alterations and dyskeratotic epithelial cells are two of the eight components of the validated Histologic Scoring System (EoEHSS) used in clinical trials (EoEHSS involves grading and staging of eosinophilic inflammation, basal zone hyperplasia (BZH), eosinophil abscess, eosinophil surface layering, intracellular spacing, surface epithelial alteration, dyskeratotic epithelial cells, and lamina propria fibrosis). Because wider use of the comprehensive EoEHSS system may be limited in clinical practice, there may be a role for such an abbreviated scoring system. Stage scores in pediatric population and grade and stage scores in adults using these parameters remain to be validated in additional studies. The link to the web-based prediction tool is https://ls2021.shinyapps.io/pre_lpf/.

The Endoscopic REFerence System (EREFS) classifies and grades five endoscopic features of eosinophilic esophagitis into inflammatory (furrows, exudates, edema) and fibrostenotic (rings and strictures) components. Remodeling or fibrostenosis features include rings, strictures, and diffuse esophageal narrowing or narrow-caliber esophagus [15] and are considered reflections of remodeling aspects of disease activity [6]. A significant association has been established with ring severity, esophageal distensibility and food impaction history. Ring severity correlate with reduced distensibility while furrows and exudates correlate with esophageal eosinophilia [16]. Upper endoscopy, however, has limited sensitivity for detecting esophageal narrowing [17–19]. Furthermore, functional consequences of subepithelial activity and remodeling or of endoscopic features may be inadequately assessed by endoscopy alone; hence the need for additional methods to assess for fibrostenosis/remodeling. Imaging in the form of barium esophagram to assess for remodeling also has its limitations, including the possibility of falsely low estimates of esophageal diameter due to inability to control the intraluminal distention force generated by swallowed barium [6].

Therein lies the role of the functional luminal imaging probe (FLIP), which uses impedance planimetry to provide an objective and accessible method of evaluating for remodeling and/or fibrostenotic disease. Biomechanical properties of the esophagus are evaluated during impedance planimetry by measuring luminal diameters and pressures during controlled volumetric distention. The probe is passed transorally during sedated endoscopy and the test can be performed within 5 min. A real time display shows a 3D view of the geometry of the esophageal lumen and, by calculating changes in cross-sectional area relative to intra-bag pressure, provides quantification of the distensibility of the esophageal body and the EGJ [20,21]. In the research space, distensibility plateau (DP) is a frequently utilized metric that reflects the fixed diameter or cross-sectional area of the esophagus that doesn’t increase in response to increasing volume or pressure. Adult EoE patients with a history of food impaction have reduced distensibility as measured by DP compared to EoE patients without history of food impaction; a lower DP is also associated with need for esophageal dilation and occurrence of food impaction with a DP diameter of <17 mm or cross-sectional area of <225 mm^2^ shown to be associated with risk for food impaction [22]. The distensibility of adult patients with EoE was also recently evaluated in a cross-sectional study of adult patients using a variety of FLIP metrics including DP, distensibility index (DI), compliance of the esophageal body and maximum EGJ diameter. Symptom duration and diagnostic delay were both negatively correlated with esophageal distensibility in patients with active disease. Patients with ≥15 eos/hpf had significantly lower DP with greater symptom duration while, in those patients in histologic remission (<15 eos/hpf), there was no significant difference in distensibility related to symptom duration [23^▪▪^]. As has been recently noted, this finding “supports the complex nature of fibroinflammatory strictures in EoE and that treatment that improves inflammation may also improve distensibility” [24^▪▪^].

In a pediatric EoE cohort, active inflammation, histologic lamina propria fibrosis, and endoscopic features of fibrostenosis were associated with decreased esophageal distensibility, as defined by “the minimal esophageal body diameter determined at maximal esophageal distension at intrabag pressure of 40 mmHg” [25]. Hoffman *et al.* also studied a pediatric cohort of 59 EoE patients and evaluated the distensibility index (DI) at the distensibility plateau, using a distensibility curve of narrowest cross-sectional-area plotted against pressure, in relation to clinical, endoscopic, and histologic features. The DI was lower in those with a clinical history of food impaction and in those with fibrostenotic features vs normal endoscopy or inflammatory features. DI did not correlate with the symptom of dysphagia or with peak eosinophils per hpf, and showed moderate inverse correlation with total EREFS score in active and inactive EoE. Of the 56% of samples that had adequate lamina propria, there was a nonstatistically significant trend in lower DI in those samples with lamina propria fibrosis. In addition, there was no correlation with DI and severity of basal zone hyperplasia (BZH) in the epithelium, which the authors suggest supported the use of BZH as a histologic marker of inflammation rather than fibrosis [26^▪▪^].

Esophageal motor function may also be impacted/driven by remodeling in the subepithelial space; e.g., hypercontractile esophageal dysmotility has been described in a case series in which eosinophilic inflammation was present only in the muscularis propria [27]. The relationship between esophageal contractile responses in the setting of secondary peristalsis induced by balloon distension during FLIP procedures and esophageal distensibility have also been evaluated in 199 adult EoE patients. Although normal contractile responses were observed in 34% of EoE patients, abnormal contractile responses (borderline, impaired/disordered, absent, and spastic-reactive) were more frequently observed in association with greater duration of symptoms, reduced esophageal distensibility, and higher ring scores (features of fibrostensosis). Interestingly, eosinophil density in esophageal biopsies was similar in EoE patients with normal and abnormal contractile responses, suggesting that they were driven by remodeling rather than ongoing inflammation [28^▪^]. These responses, along with esophageal distensibility during evaluation by FLIP, have recently been incorporated into a physio-mechanical model of esophageal function in EoE, that identifies seven phenotypes distinguished by symptom duration and EREFS scores. Patients with the spastic-reactive fibrostenotic and nonreactive fibrostenotic phenotypes, in particular, had greater symptom duration and diagnostic delay compared to those with normal phenotype. Endoscopic features of fibrostenosis including ring score and stricture severity were also most prevalent in those with the fibrostenotic phenotypes [29^▪^].

Limitations of FLIP studies include the variable modes and complexity of techniques used to analyze FLIP metrics [24^▪▪^,^30^]. Furthermore, metrics described in those studies may not reflect parameters readily available to practitioners who typically use real-time displays of balloon pressure along with diameter and software-calculated distensibility index along the length of the esophageal body and EGJ [21].

Luminal markers of remodeling have also been investigated. Muir *et al.* utilized the esophageal string test in which a string attached to a swallowed capsule allows for sampling of esophageal epithelial cells, inflammatory cells, and luminal secretion. They looked at whether previously described eosinophil associated proteins (EAPs) that could be captured by EST (EDN, EPX, MBP-1, Gal-10, CCL26) and periostin, an extracellular matrix protein upregulated in fibrosis, correlated with histologic or endoscopic features of remodeling in 40 pediatric patient with active and inactive EoE. Luminal EAPs correlated with biopsy tissue EAPs, mucosal eosinophils, and endoscopic and histologic features of EoE, including markers of epithelial-mesenchymal transition and negatively with esophageal distensibility as measured with FLIP. Periostin also correlated with EAPs, endoscopic and histologic features of EoE including epithelial-mesenchymal transition, although it did not correlate with distensibility [31^▪^]. Previous studies have shown lack of correlation between serum periostin and disease activity [32].

## PATHOGENESIS OF FIBROSIS AND REMODELING IN EOSINOPHILIC ESOPHAGITIS

One of the primary effector cells of subepithelial fibrosis is the fibroblast, which is responsible for deposition of extracellular matrix (ECM) proteins and, in the Th2 cytokine context of EoE, can secrete the eosinophil chemoattractant eotaxin-3 [33]. Transforming growth factor-β (TGF-β), a critical pro-fibrotic cytokine, is produced by infiltrating eosinophils and mast cells, and is present in a latent form in the ECM [34]. TGF-β induces fibroblast activation and secretion of ECM proteins such as collagen and fibronectin [35^▪▪^]. TGF-β signaling has been shown to be associated with esophageal fibrosis and angiogenesis in a mouse model of EoE [36].

Epithelial-mesenchymal transition and epithelial acquisition of myofibroblast characteristics including the ability to contract, migrate, and participate in ECM protein synthesis and deposition in response to TGFβ (most robust effect on epithelial collagen production) or TNFα (most potent effect on cell migration and contraction) has been described [37]. A study using primary esophageal fibroblasts from EoE patients also demonstrated the importance of tissue stiffness on fibroblast behavior, showing that matrix stiffness increases TGF-β-mediated gene expression and activation of fibroblasts to activated myofibroblasts [38]. Comparative esophageal ECM proteomics of normal vs. EoE patients has demonstrated that the ECM in severe EoE alters normal fibroblast function and protein expression of collagen I, fibronectin, and αSMA through thrombospondin-1 [39^▪▪^].

The importance of esophageal fibroblast-epithelial cross-talk in EoE-associated fibrogenesis is further shown in work identifying a potential role for an increase in epithelial expression of TGF-β and of LOX, which can cross-link collagen, in EoE patients with fibrostenotic symptoms and endoscopic signs of fibrosis in response to fibroblast-derived TNF-α [40]. More recently, mouse models of EoE show that altered epithelial biology in the aging esophagus may contribute to EoE-associated fibrosis, possibly via upregulation of soluble factors/cytokines. Although the correlation of these mouse-based experiments with distensibility in the human esophagus remains to be defined, these findings identify possible age-associated factors that may contribute to fibrotic remodeling, and that may be of particular relevance when considering pediatric versus adult EoE [41].

Fibroblast cross-talk with endothelial cells has also been implicated in the pathogenesis of fibrostenosis in EoE via loss of endothelial TSPAN12 [42^▪▪^]. Using esophageal biopsies from pediatric and adult subjects across 11 sites in the Consortium of Eosinophilic Gastrointestinal Disease Researchers and in 2 independent replication cohorts, Shoda *et al.* found that nearly 50% of the patients had fibrostenotic EoE (endoscopic rings, stricture, and/or a history of dilation), and they identified TSPAN12, a member of the EoE transcriptome, as the gene most correlated with fibrostenosis. TSPAN12 expression was significantly lower in fibrostenotic vs. nonfibrostenotic EoE and, in addition to correlating with endoscopic, histologic, and molecular features of EoE, TSPAN12 also correlated with fibrostenotic features (i.e., TSPAN12 correlated directly with esophageal diameter and inversely with lamina propria fibrosis and with ECM-related genes including collagens and MMPs). Single-cell RNA sequencing showed that TSPAN12 was most significantly enriched in the endothelium of esophageal tissue compared to other cell populations; this finding was confirmed in *in vitro* studies of various cell types. scRNA-seq also showed that TSPAN12 expression was lower in active EoE vs. control samples. *In vitro* studies further showed that interleukin (IL)-13 [a cytokine with a central role in the pathogenesis of EoE, including recapitulating the esophageal transcriptome from EoE biopsy specimens [43]] reduces endothelial TSPAN12 expression, leading to an impaired barrier and elaboration of profibrotic mediators that ultimately increase ECM protein production by fibroblasts [42^▪▪^].

Wen *et al.* identified inflammatory tissue T cells in EoE using a combination of flow cytometry, bulk CD3^+^ RNA-seq and single-cell RNA-seq [44]. Single-cell RNA sequencing (scRNA-seq) was applied to biopsies from EoE patients with a spectrum of disease activity and from healthy controls. They identified eight T cell subclasses (T1–T8) in the esophageal tissue, including CD8^+^ clusters T1–6 and, importantly, two CD4^+^ clusters (T7 and T8) that were enriched in EoE patients with active disease and which represented a population similar to CD4^+^ Tregs with a regulatory associated phenotype (T7) and a population enriched for CD4^+^ Th2 cells (T8) producing type 2 cytokines (IL13 and IL5) transcripts. T8 was notably absent from healthy controls and T8 comprised only 0.4% of T cells from subjects in remission; these findings implicate the T8 cluster in EoE disease development [44,45].

LIGHT (tumor necrosis factor superfamily member 14) is a TNF-related cytokine that contributes to tissue remodeling and induces inflammatory gene expression in lung fibroblasts and epithelial cells. Increased LIGHT production by T cells in EoE promotes differentiation of esophageal fibroblasts toward an inflammatory phenotype [46]. Manresa *et al.* used the publicly available T cell database to show that LIGHT was present in several of the T cell clusters, including the majority of IL13^+^IL5^+^CD4^+^ T-helper type 2 cells in the human esophagus. They also showed increased expression of LIGHT+ cells in the lamina propria and epithelium of esophageal biopsies of active EoE and, in the epithelium, an increase in CD3^+^, LIGHT^+^ cells. In vitro studies showed that LIGHT induced an inflammatory profile in esophageal fibroblasts (with expression of ICAM-1, IL-32, TNFSF15, CXCL5, IL-33, IL-34) and mediated fibroblast-eosinophil interactions via ICAM-1. T cell-derived LIGHT also modulated the TGF-β-induced myofibroblast transcriptome/phenotype (ACTA2, MYH11, TAGLN, FLNA, ACTG2) to an inflammatory type of myofibroblast [46].

More recent work has focused on the role of LIGHT receptors, herpes virus entry mediator (HVEM) and lymphotoxin-beta receptor (LTβR) on esophageal fibroblasts. HVEM and LTβR are stably expressed in esophageal fibroblasts, while HVEM expression increases in response to TGF-β. These fibroblast receptors mediate a unique gene expression profile in response to LIGHT, including ICAM-1 and IL-34, and work in concert to tether fibroblasts to eosinophils in co-culture. Spatial transcriptomics via RNA *in situ* hybridization reveals a predominantly homeostatic VIM^+^WNT2B^+^ fibroblast population in the normal esophagus lamina propria, and a shift to an inflammatory VIM^+^ICAM-1^+^IL-34^+^ population of fibroblasts in active EoE. Overall, this work suggests that, in addition to mediating fibrosis, EoE fibroblasts via T cell mediators such as LIGHT, may also be important in coordinating the inflammatory response [47^▪▪^].

Expression of epithelial plasminogen activator inhibitor-1 (PAI-1), a serine protease inhibitor induced by TGF-β1, is increased in pediatric EoE. PAI-1 promotes fibrotic and myofibroblast gene expression in esophageal fibroblasts, and PAI-1 levels correlate with lamina propria fibrosis [48]. More recently, PAI-1 together with basal zone hyperplasia has been shown to help predict esophageal rigidity in pediatric EoE [49^▪^]. In a study of 45 children (32 with and 13 without EoE), PAI-1 immunostaining was increased in patients with active EoE versus those with inactive EoE and control patients, and PAI-1 immunostaining positively correlated with basal zone hyperplasia. In addition, proximal esophageal compliance as assessed by EndoFLIP was lower in EoE patients than controls, and a threshold compliance of 2.6% ml/mmHg was found to predict EoE vs. non-EoE. A negative correlation was identified between PAI-1 expression and esophageal compliance (higher esophageal compliance reflects a normal esophageal functional state). Multivariate logistic regression modeling demonstrated that a composite score of PAI-1 immunostaining score and basal zone hyperplasia predicted esophageal compliance [49^▪^]. This work, along with work regarding esophageal string test described above, shows promise in the goal of identifying molecular markers of fibrosis. Additional studies are needed to translate the utility of these findings to clinical practice.

## THERAPY OF REMODELING

Therapy for EoE includes elimination diets, medications (PPIs, topical corticosteroids, most recently biologic dupilumab), and esophageal dilation. Results regarding reversibility of esophageal remodeling in response to dietary interventions and medications have been mixed, with results differing in pediatric versus adult populations and dependent on duration and type of therapy. Earlier studies in the pediatric population with topical steroids and/or elimination diets showed some improvement in subepithelial fibrosis scores [50,51], profibrogenic cytokine gene expression [52], and reduction in epithelial-mesenchymal transition (wherein epithelial cells acquire myofibroblast characteristics) [53] with control of inflammation and reduction or normalization of epithelial eosinophil number. Earlier results in adults treated with topical steroids were more mixed, in part due to different types of formulations and/or durations being investigated and different measures of fibrosis, including immunostaining for fibosis and radiographic tools utilized across studies [54–57]). More recently in EoE patients between ages 11 and 40, treatment with a budesonide oral suspension resulted in significant improvement in all EREFS parameters except strictures (i.e., there were significant improvements in edema, rings, exudates, and furrows), as well as significant improvements in tissue eosinophilia and symptoms [58].

A study of 18 adult patients with eosinophilic esophagitis showed improvements in esophageal distensibility (reflected by an increase in esophageal distensibility plateau median of 13.9 mm to 16.8 mm) in response to medical therapy with topical steroids or proton-pump inhibitors and elimination diet without dilation. Improvements in distensibility also correlated with improvement in endoscopic ring score, an EREFS feature of fibrostenosis, and were related to improvements in symptoms [59]. The effect of dupilumab (a recently FDA approved human monoclonal antibody that blocks signaling of interleukin-4 and interleukin-13) on esophageal distensibility has been investigated as part of an exploratory endpoint. Compared to placebo, patients treated with dupilumab showed improvement, although not normalization, of distensibility by 18% at week 12 [60]. A retrospective study of EoE patients refractory to treatments with standard modalities, most with fibrostenotic disease (85% had prior esophageal dilations), showed post dupilumab histology response rates of 80% and a significant increase in esophageal diameter (before dilation) from 13.9 to 16 mm [61^▪^].

Dilation, if done carefully, can offer immediate relief from strictures safely, with a low risk of perforation, although postprocedure chest pain is nearly universal. Furthermore, dilation does not address the underlying inflammation. A retrospective chart review of EoE patients with severe strictures (esophageal diameter <10 mm) identified initial stricture diameter and histologic remission as factors significantly associated with achieving a diameter ≥15 mm after treatment with medical or dietary therapy in conjunction with esophageal dilations [62].

## CONCLUSION

Esophageal remodeling in EoE adds to the clinical burden of this disorder, manifesting with dysphagia and food impaction. Understanding of the mechanisms of subepithelial fibrosis contributing to esophageal remodeling in EoE has evolved over the past few years, led in part by some of the studies highlighted in this review. Improved understanding of the pathogenesis and diagnostic assessment of remodeling enhances clinical care, as it improves our understanding of the effects of current therapeutic interventions and paves the way for development of newer targeted therapeutics. Finally, the ability to accurately diagnose remodeling with histopathology, endoscopy, and newer tools such as impedance planimetry can lead to improved, more personalized, and mechanistically-targeted care of this increasingly recognized disorder.

## Acknowledgements

*None.*

### Financial support and sponsorship

*This work was supported by National Institute of Diabetes and Digestive and Kidney Diseases Grant R01-DK118065*

*Funding: R01DK118065.*

### Conflicts of interest

*There are no conflicts of interest.*

## REFERENCES AND RECOMMENDED READING

Papers of particular interest, published within the annual period of review, have been highlighted as:

▪ of special interest
▪▪ of outstanding interest
